# Vitamin D Deficiency and Depressive Symptomatology in Psychiatric Patients Hospitalized with a Current Depressive Episode: A Factor Analytic Study

**DOI:** 10.1371/journal.pone.0138550

**Published:** 2015-09-23

**Authors:** Roland von Känel, Nasser Fardad, Nadine Steurer, Nicole Horak, Esther Hindermann, Franz Fischer, Katharina Gessler

**Affiliations:** 1 Department of Psychosomatic Medicine, Clinic Barmelweid, Barmelweid, Switzerland; 2 Department of Neurology, Bern University Hospital, Inselspital, and University of Bern, Bern, Switzerland; 3 Department of Clinical Research, University of Bern, Bern, Switzerland; 4 Psymeta GmbH, Schafisheim, Switzerland; Maastricht University, NETHERLANDS

## Abstract

**Background:**

Low vitamin D levels have been associated with depressive symptoms in population-based studies and non-clinical samples as well as with clinical depression. This study aimed to examine the association of vitamin D levels with the severity and dimensions of depressive symptoms in hospitalized patients with a current episode of depression taking into account confounding variables.

**Methods:**

We investigated 380 patients (mean age 47±12 years, 70% women) who were consecutively hospitalized with a main diagnosis of an ICD-10 depressive episode. All patients self-rated depressive symptom severity with the Hospital Anxiety and Depression Scale (HADS-D), the Beck Depression Inventory-II (BDI-II), and the Brief Symptom Inventory. A principal component analysis was performed with all 34 items of these questionnaires and serum levels of 25-hydroxyvitamin D3 (25-OH D) were measured.

**Results:**

Vitamin D deficiency (<50 nmol/l), insufficiency (50–75 nmol/l), and sufficiency (>75 nmol/l) were present in 55.5%, 31.8% and 12.6%, respectively, of patients. Patients with vitamin D deficiency scored higher on the HADS-D scale and on an anhedonia symptom factor than those with insufficient (p-values ≤0.023) or sufficient (p-values ≤0.008) vitamin D. Vitamin D deficient patients also scored higher on the BDI-II scale than those with sufficient vitamin D (p = 0.007); BDI-II cognitive/affective symptoms, but not somatic/affective symptoms, were higher in patients with vitamin D deficiency (p = 0.005) and insufficiency (p = 0.041) relative to those with sufficient vitamin D. Effect sizes suggested clinically relevant findings.

**Conclusions:**

Low vitamin D levels are frequent in hospitalized patients with a current episode of depression. Especially 25-OH D levels <50 nmol/l were associated with cognitive/affective depressive symptoms, and anhedonia symptoms in particular.

## Introduction

Systematic reviews and meta-analyses of population-based cross-sectional and prospective cohort studies have shown that low serum levels of 25-hydroxyvitamin D3 (25-OH D) are associated with depressive symptoms and clinical depression, respectively [1,2]. Clinical Practice Guidelines from the US Endocrine Society define vitamin D deficiency as 25-OH D less than 50 nmol/l, vitamin D insufficiency as 25-OH D between 50 and 75 nmol/l, and vitamin D sufficiency as 25-OH D greater than 75 nmol/l [3]. However, it should be noted that the issue of accepted clinical cut-offs for vitamin D deficiency is somewhat controversial; for instance, the US Institute of Medicine report defines vitamin D deficiency as 25-OH D less than 40 nmol/l [4]. With the cut-offs recommended by the US Endocrine Society applied to a national representative sample of young adults, the odds ratio for a current depressive episode was 1.8-fold higher in individuals with deficient vitamin D when compared to those with sufficient vitamin D [5]. Moreover, vitamin D supplementation with at least 800 I.U. daily improves depression to a similar extent as antidepressant medications [6] and vitamin D supplementation may augment the effect of antidepressants in patients with major depressive disorder [7]. Therefore, there is now ample evidence for the assumption that vitamin D deficiency plays a role at least in persons with clinically relevant depressive symptoms. Plausible neurobiological and neuroendocrine mechanisms have been proposed for this link, including a role of vitamin D in brain areas processing depressive mood [8], in serotoninergic and dopaminergic function [9,10], and in constraining systemic inflammation being associated with depression [11].

Surprisingly few studies have investigated the relation between vitamin D deficiency and depressive symptom severity in patients with a psychiatric diagnosis of depression. One study we could track showed increased scores for the Hamilton Depression Rating Scale in vitamin D deficient outpatients with a major depressive disorder without psychosis, although no adjustment was made for covariates [7]. This is crucial, because several risk factors for vitamin D deficiency have been identified some of which are also associated with depression; these include ageing, female sex, dark skin pigmentation, winter season, obesity, impaired liver function, no use of vitamin D supplements, antidepressant and anticonvulsant medications, and psychiatric comorbidity like anxiety and eating disorders [5,12–15]. A range of physical medical conditions, including cardiovascular disease, liver disease and renal disease may also be associated with low vitamin D levels [3,4].

To our knowledge, no study to date has examined a relation between vitamin D and the severity of depressive symptoms in hospitalized patients with clinical depression. Such a relationship is of particular clinical importance because a need for hospitalization may indicate more severe illness with ultimate implications of vitamin D deficiency for multicomponent antidepressant treatment. This even more so, as antidepressant effects of vitamin D supplementation can particularly be expected in patients with clinically significant depressive symptoms [16]. However, not all clinically depressed patients may have deficient or insufficient vitamin D, so uncritical substitution may pose harm to those depressed patients with sufficient vitamin D. For instance, the US Endocrine Society defines a safety margin to minimize the risk of hypercalcemia as 25-OH D equal to 250 nmol/l [3]. The US Institute of Medicine report already raises concerns about potential adverse effects with 25-OH D equal to 125 nmol/l [4]. Side effects that may typically occur with 25-OH D levels above 375 nmol/l are hypercalcemia, hypercalciuria, and hyperphosphatemia with the associated soft-tissue and vascular calcifications and nephrolithiasis in the long term [12]. Furthermore, clinical depression can also be induced by hypercalcemia due to hypervitaminosis D [17].

Depression is a heterogeneous concept regarding its symptom dimensions with studies showing that, for instance, cognitive, somatic, and neurovegetative depressive symptoms differently relate to a range of health outcomes, including biological ones [18–20]. A recent study reported vitamin D deficiency or insufficiency to be associated with specific psychopathology in schizophrenia patients, including grandiosity, social anhedonia and irregular speech [21]. Therefore, we also computed an exploratory principal component analysis using items from three widely used self-rated depressive symptom scales to possibly identify depressive symptom factors with a particularly strong relationship with vitamin D deficiency, which, to our knowledge, has not previously been tested.

The aims and novel aspects of this study were to investigate the relationship between vitamin D status and both the severity and dimension of depressive symptomatology in hospitalized patients with psychiatric depression applying three validated depressive symptom scales. We specifically hypothesized that patients with a current depressive episode plus vitamin D deficiency (<50 nmol/l) would show higher levels on depressive symptom scales, and possibly depressive symptom factor scores, when compared with depressed patients with sufficient (<75 nmol/l) and also insufficient (50–75 nmol/l) 25-OH D serum levels, even after adjustment for important confounding variables of this relationship.

## Materials and Methods

### Study participants and design

The 380 patients who participated in this study were consecutively hospitalized between January 2012 and December 2014 with a primary diagnosis of an ICD-10 depressive episode without psychosis at the Department of Psychosomatic Medicine, Clinic Barmelweid, Switzerland, to undergo inpatient psychosomatic rehabilitation. Within the Swiss healthcare system, inpatient rehabilitation for adults is legally covered by insurance granted that several eligibility criteria are met. These include insufficient effectiveness of outpatient (psycho)therapy, a need for distance from the family and/or job environment, functional impairment with insufficient maintenance of activities of daily living and mobility, problem behaviors that cannot be controlled by the patient (e.g., severe avoidance behavior), sustained inability to work or risk of becoming disabled, and motivation and ability to participate in a multimodal (aka interdisciplinary or multicomponent) treatment program.

**Fig 1:**
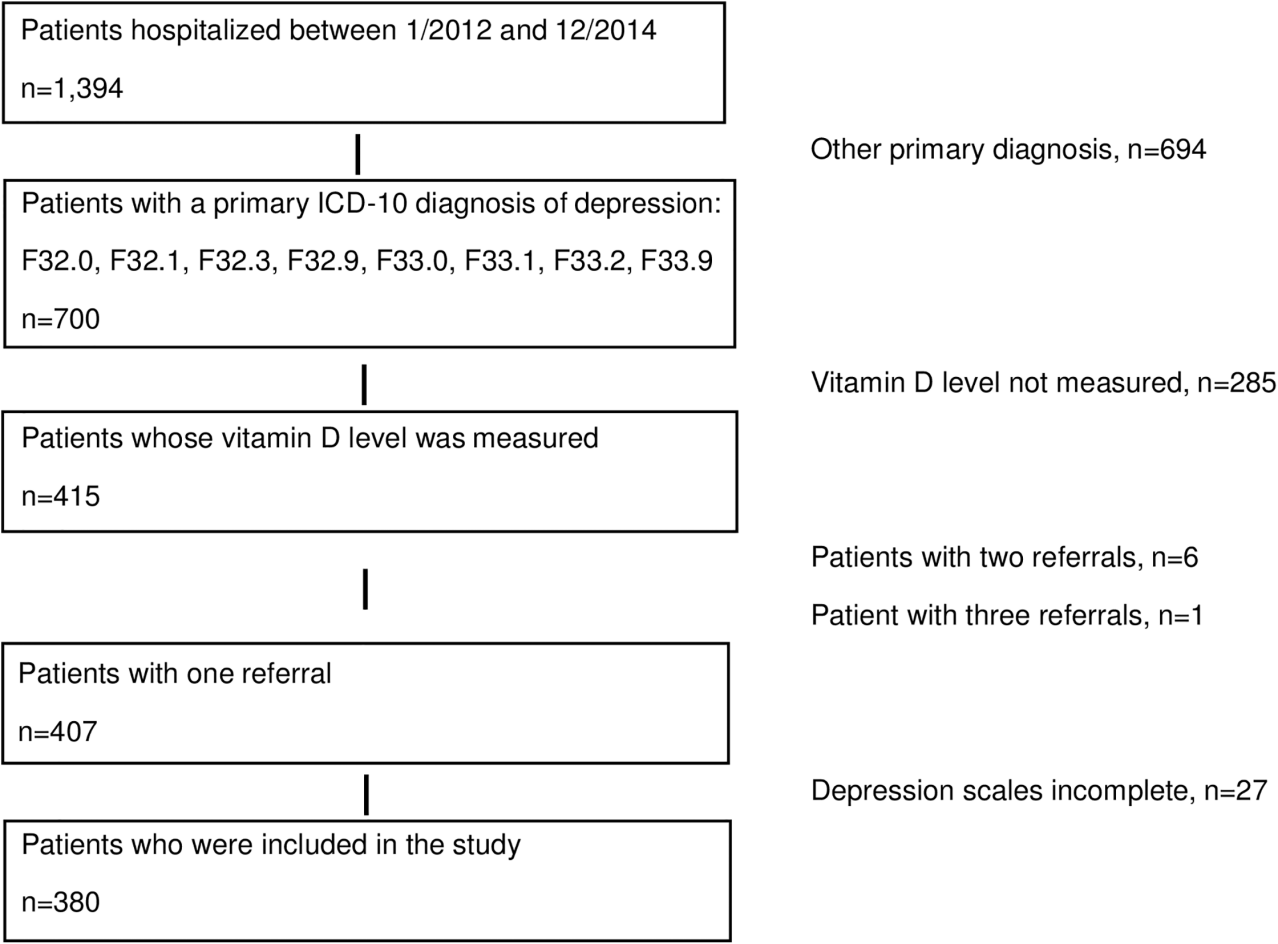
Flow chart of the included 380 patients with a depressive episode.

Fig 1 depicts the flowchart of the patient recruitment. Specific inclusion criteria were a main diagnosis of a first-time or recurrent mild (F32.0/F33.0), moderate (F32.1/F33.1), severe (F32.2/F33.2) or unspecified (F32.9/F33.9) depressive episode and complete data for psychometric questionnaires and vitamin D status. For patients who, during the observation period, were hospitalized more than once, we used data from the first hospitalization. Because of well-known high rates of comorbidities in depressed inpatients, other mental disorders and physical diseases were not exclusion criteria.

The diagnosis of a depressive episode, including its severity and recurrence, and of other major psychiatric diagnoses and physical diseases were made by one medical coder based on discharge reports prepared by a board-certified psychiatrist /psychosomatic medicine specialist. The medical coder has been certified by an accredited agency training coders to make ICD-10 diagnoses based on Swiss Diagnosis Related Groups (DRG), a system to classify hospital cases into groups for reimbursement, which became effective in January 2012. Moreover, federal regulations (represented by the National Society for Quality Development in Hospitals) require standardized assessments of demographic and psychometric data for quality control of inpatient psychosomatic treatment. As part of clinical routine, a fasting morning chemistry screening panel is done at hospital entry in virtually every patient, whereas vitamin D status has increasingly been determined over the last years in our clinic, systematically so since 2014.

All psychometric and laboratory data were obtained within the first three days of the hospitalization and collected from hospital charts and electronic files retrospectively for the purpose of this study between December 2014 and March 2015. All patients agreed their data could be used for quality control of the inpatient treatment program. Patient records and information was anonymized and de-identified prior to analysis. The Ethics Committee for Northwest/Central Switzerland approved the study protocol.

### Psychometric assessment

We measured the severity of depressive symptoms with three self-rated instruments validated in German. Patients received a personal login to complete web-based versions of questionnaires. Master level psychology students supervised by a clinical psychologist reviewed questionnaire data and encouraged patients to fill in items left blank, so to meet standards for quality control required by federal regulations.

#### Hospital Anxiety and Depression Scale

The 7-item depression subscale of the Hospital Anxiety and Depression scale (HADS-D) covers the severity of depressive symptoms in the previous week [22,23] and has been widely used in psychiatric populations [24]. The items exclude somatic/affective symptoms of depression to avoid any bias with symptoms from coexisting medical conditions. Each item is rated on a 4-point Likert scale ranging from 0 ("mostly") to 3 ("not at all"), yielding a total score between 0 and 21. A score ≥ 8 defines the threshold for clinically relevant depressive symptoms. In the current sample, Cronbach’s alpha was 0.82, indicating good reliability of the HADS-D.

#### Beck Depression Inventory-II

The 21-item Beck Depression Inventory-II (BDI-II) covers the severity of depressive mood over the last two weeks, including today [25,26]. Each item is scored with a value between 0 and 3, yielding a total score between 0 and 63. A score ≥ 14 defines the threshold for clinically relevant depressive symptoms. For clinical samples, the BDI-II items can be divided into cognitive/affective and somatic/affective subscales, for instance, based on the model by Beck et al [25]; the validity of these subscales has recently been reevaluated [27]. In the current sample, Cronbach’s alpha was 0.91, indicating excellent reliability of the BDI-II total score. The cognitive/affective and somatic/affective subscales showed good reliabilities with a Cronbach's alpha of 0.88 and 0.84, respectively.

#### Brief Symptom Inventory

The 6-item depression subscale of the Brief Symptom Inventory (BSI-D) covers the severity of depressive symptoms in the previous seven days [28,29]. The patients rated the intensity of each item on a 5-point Likert scale ranging from 0 ("not at all") to 4 ("very strong"), yielding a total score between 0 and 24. In the current sample, Cronbach’s alpha was 0.84, indicating good reliability of the BSI-D.

### Vitamin D status

Vitamin D (25-OH D) was measured in serum by certified commercial laboratories using liquid chromatography-mass spectrometry between January 2012 and December 2013 and, starting January 2014, using an enzyme-linked fluorescent assay. Lower limits of detection are 10 nmol/l for the first assay and 20.3 nmol/l for the second one. To account for the different techniques, the applied vitamin D assay method was dummy coded and used as a covariate in the multivariate analysis. Following previous guidelines for the definition of vitamin D status, 25-OH D concentrations >75 nmol/l were considered sufficient vitamin D. Levels between 50 and 75 nmol/l and <50 nmol/l of 25-OH D were defined insufficient and deficient vitamin D levels, respectively [3].

### Assessment of covariates

Age in years and sex were noted. Following the patients' regional origin, skin pigmentation was dummy coded as dark (Southern and Eastern Europe) or light (Middle Europe) [30]. We defined the six months with the least (November to April) and the most (May to October) statistical sunshine hours in Switzerland as winter and summer seasons, respectively [31]. Medical staff measured height and weight with patients wearing light clothing. Obesity was defined by a body mass index ≥ 30 kg/m^2^ and used as a covariate.

To control for inflammatory processes, C-reactive protein levels were measured in serum (C-reactive Protein (Latex), Cobas Integra 400 plus, Roche Diagnostics, Mannheim, Germany) and dummy coded as normal (≤5 mg/L) or elevated (>5 mg/L). One missing value for C-reactive protein was replaced by the sample mean. To account for insufficient production of vitamin D due to any liver affection, catalytic activity of transaminases was determined in serum (Cobas Integra). Activity of alanine aminotransferase (aspartate aminotransferase in case of missing alanine aminotransferase) was dummy coded as normal or elevated (>50 U/L for men; >35 U/L for women). One missing value was replaced by the sample mean.

We grouped comorbid psychiatric disorders following ICD-10 criteria as anxiety disorders (F40, F41), somatoform disorders (F45), posttraumatic stress disorder (PTSD, F43.1), and eating disorders (F50). We noted the number of antidepressants, neuroleptics, pain medications and anticonvulsants at hospital entry. These medications may point to illness severity and/or interact with vitamin D metabolism.

### Statistical analysis

Data were analyzed using PASW 18.0 statistical software package (SPSS Inc., Chicago, IL, USA) with p-level < 0.05 (two-tailed). We first computed a principal component analysis with oblimin rotation using standardized z-scores of all 34 items from the HADS-D, BDI-II, and BSI-D to identify Bartlett factor scores for depressive symptoms with Eigenvalues > 1, applying parallel analysis (Monte Carlo simulation) [32]. We then used multivariate analysis of variance (MANOVA) and of covariance (MANCOVA) to compare the three vitamin D status groups (deficiency, insufficiency, sufficiency) on scores of depressive symptom scales and factors, respectively, applying Roy's largest root. We defined covariates a priori, based on their known associations with vitamin D deficiency and/or depression [5,12–15]; these were age, sex, season, skin pigmentation, obesity, elevated levels of C-reactive protein and/or transaminases, anxiety disorders, PTSD, somatoform disorders, eating disorders, use of vitamin D supplements, the number of antidepressants (0 vs. 1 vs. 2), neuroleptics (0 vs. ≥1), and pain medications (0 vs. 1 vs. ≥2), use of anticonvulsants (yes/no), recurrent depressive episode, and vitamin D assay technique. Pearson correlations quantified the bivariate association between two continuous variables. Group differences for categorical variables were calculated with Pearson chi-square test or Fisher’s exact test. The effect sizes for the AN(C)OVAs were expressed as eta-squared (η^2^) with values of 0.01, 0.06, and 0.14 indicating small, moderate, and large effects, respectively [33]. Effect sizes for differences in mean values were expressed as Cohen's *d*, with values of 0.2, 0.5, and 0.8 indicating small, moderate, and large effects, respectively [33].

## Results

### Participant characteristics

The study sample of 380 patients with a main diagnosis of an ICD-10 depressive episode was middle aged (47.2±12.9 years) and comprised of about two-fold more women than men (69.5 vs. 30.5%). Psychiatric co-morbidity was substantial with an anxiety disorder in 24.7%, PTSD in 7.4%, a somatoform disorder in 25.0%, and an eating disorder in 7.1%. Moreover, 32.6% were obese. Two-fold as many patients suffered from a recurrent (F33) than from a first-time (F32) depressive episode (65.0 vs. 35.0%). The current episode was mild in 21 (5.5%) cases (F32.0, F33.0), moderate in 252 (66.3%) cases (F32.1, F33.1), severe in 82 (21.6%) cases (F32.2, F33.2), and unspecified in 25 (6.6%) cases (F32.9, F33.9). Regarding comorbid physical medical conditions, 19 patients had cardiovascular disease (ischemic heart disease, hypertensive heart disease, peripheral vascular disease), one had hypertensive heart disease plus chronic kidney disease, stage 3, and two had liver cirrhosis.

### Exploratory factor analysis for depressive symptoms

The mean ± SD value was 12.53 ± 4.35 (range 1–21) for the HADS-D, 30.48 ± 11.68 (range 3–58) for the BDI-II, and 11.57 ± 5.83 (range 0–24) for the BSI-D. The depressive symptom scales correlated strongly with each other (all p-values <0.001): HADS-D and BDI-II: r = 0.73; HADS-D and BSI-D: r = 0.68; and BDI-II and BSI-D: r = 0.84. There also was a correlation between the BDI-II cognitive/affective and somatic/affective symptom subscales (r = 0.68, p<0.001). Based on a HADS-D score ≥8 and a BDI-II score ≥14, clinically relevant depressive symptoms were reported in 324 (85.3%) and 352 (92.6%) of patients, respectively.

The comprehensive principal component analysis using all 34 items from the HADS-D, BDI-II and BSI-D revealed 36 factorial units to explain 100% of the variance by definition. Six factorial units had an Eigenvalue >1. Parallel analysis yielded a solution with three factors to be retained, together explaining 48.7% of the variance. Further inspection of the communalities output showed four items (i.e., BDI-II #10/crying, BDI-II #11/agitation, BDI-II #16/changes in sleeping pattern, BDI-II #18/changes in appetite) with correlations <0.30, indicating low fit with the other items in the three factors. To improve the efficiency of the factor analysis, these items were deleted to obtain a final three-factor solution that explained 52.8% of the variance. For illustrative purposes, Table 1 shows the individual items from the three depressive symptom scales with loadings >0.4 on the three factors termed "Negative Affect", "Anhedonia", and "Inhibition". All factors were normally distributed and correlated moderately with each other (all p-values <0.001): negative affect and anhedonia: r = 0.51; negative affect and inhibition: r = 0.29; anhedonia and inhibition: r = 0.34.

**Table 1:** Summary of the rotated three-factor solution with item loadings.

Scale/item	Content	Factor 1	Factor 2	Factor 3
		Negative affect	Anhedonia	Inhibition
BDI-II #7 c/a	Self-dislike	0.794		
BDI-II #3 c/a	Sense of failure	0.785		
BDI-II #8 c/a	Self-accusations	0.783		
BSI #50	Feelings of worthlessness	0.773		
BDI-II #5 c/a	Guilty feelings	0.771		
BDI-II #14 c/a	Worthlessness	0.721		
BDI-II #6 c/a	Punishment feelings	0.621		
BSI #35	Feeling hopeless about the future	0.588		
BDI-II #9 c/a	Suicidal thoughts or wishes	0.569		
BSI #9	Thoughts about ending your life	0.552		
BSI #16	Feeling lonely	0.515		
BDI-II #2 c/a	Feling discouraged about the future	0.477		
BDI-II #17 s/a	Irritability	0.401		
HADS #2	Enjoy the things I used to enjoy		0.861	
HADS #4	Can laugh and see the funny things of life		0.832	
HADS #14	Can enjoy a good book/radio/Tv program		0.742	
BDI-II #4 c/a	Loss of pleasure		0.700	
HADS #6	Feel cheerful		0.673	
BDI-II #12 c/a	Loss of interest		0.595	
BSI #18	Feeling no interest in things		0.499	
HADS #12	Look forward with enjoyment to things		0.488	
HADS #10	Lost interest in my appearance		0.442	
BDI-II #1 c/a	Feeling sad		0.437	
BDI-II #21 s/a	Loss of interest in sex		0.412	
BDI-II #20 s/a	Tiredness or fatigue			0.726
BDI-II #15 s/a	Loss of energy			0.616
BDI-II #19 s/a	Concentration difficulty			0.520
HADS #8	Slowed down			0.511

Only item loadings >0.4 are shown. BDI-II, Beck Depression Inventory-II (c/a, cognitive/affective item; s/a, somatic/affective item); BSI, Brief Symptom Inventory; HADS, Hospital Anxiety and Depression Scale

### Associations of vitamin D status with covariates

Table 2 shows that vitamin D status was significantly associated with season and the use of vitamin D supplements and antidepressants. As expected, more patients with vitamin D deficiency than with insufficient (p<0.001) or sufficient (p = 0.010) vitamin D were hospitalized during the winter season. Patients with vitamin D deficiency used less frequently vitamin D containing supplements than those with sufficient (p<0.001) or insufficient (p = 0.008) vitamin D. Patients with sufficient vitamin D were prescribed a greater number of antidepressant medications than those with vitamin D deficiency (p = 0.004).

**Table 2:** Associations between vitamin D status and covariates in 380 patients.

Variables	Deficiency	Insufficiency	Sufficiency	P-value
	25-OH D <50 nmol/l	25-OH D = 50–75 nmol/l	25-OH D > 75 nmol/l	
	n = 211, 55.5%	n = 121, 31.8%	n = 48, 12.6%	
Age (yrs)	47.53 ± 13.50	47.10 ± 12.27	46.01 ± 12.08	0.761
Female sex, n (%)	142 (67.3)	88 (72.7)	34 (70.8)	0.572
Winter season, n (%)	118 (55.9)	41 (33.9)	17 (35.4)	<0.001
Dark skin pigmentation, n (%)	48 (22.7)	19 (15.7)	9 (18.8)	0.295
Obesity, n (%)	77 (36.5)	37 (30.6)	10 (20.8)	0.095
Elevated liver enzymes, n (%)	21 (10.0)	12 (9.9)	5 (10.4)	1.000
Elevated CRP, n (%)	38 (18.0)	19 (15.7)	5 (10.4)	0.427
Anxiety disorder, n (%)	57 (27.0)	30 (24.8)	7 (14.6)	0.197
PTSD, n (%)	19 (9)	7 (5.8)	2 (4.2)	0.461
Somatoform disorder, n (%)	55 (26.1)	27 (22.3)	13 (27.1)	0.703
Eating disorder, n (%)	10 (4.7)	11 (9.1)	6 (12.5)	0.093
Vitamin D supplement, n (%)	14 (6.6)	19 (15.7)	12 (25.0)	0.001
Antidepressants				
No antidepressants	63 (29.9)	27 (22.3)	15 (31.3)	0.009
1 antidepressant	118 (55.9)	67 (55.4)	17 (35.4)	
2 antidepressants	30 (14.2)	27 (22.3)	16 (33.3)	
Neuroleptic use, n (%)	48 (22.7)	28 (23.1)	10 (20.8)	0.947
Anticonvulsant use, n (%)	31 (14.7)	16 (13.2)	11 (22.9)	0.270
Pain medications				
No pain medications	110 (52.1)	67 (55.4)	28 (58.3)	0.507
1 pain medication	66 (31.1)	42 (34.7)	14 (29.2)	
≥ 2 pain medications	35 (16.6)	12 (9.9)	6 (12.5)	
Recurrent depression, n (%)	132 (62.6)	84 (69.4)	31 (64.6)	0.450

Values are means±SD or absolute numbers (percentages). CRP, C-reactive protein; PTSD, posttraumatic stress disorder

### Vitamin D status and severity of depressive symptoms

#### Unadjusted models

There was a significant group effect for vitamin D status in the MANOVA analysis with all three depressive symptom scales (F_3,376_ = 4.56, p = 0.004; η^2^ = 0.035) and also in the MANOVA analysis with all three depressive symptom factor scores (F_3,376_ = 4.85, p = 0.003; η^2^ = 0.037) as the dependent variables. As shown in Table 3, there were significant group differences in the HADS-D score (p = 0.006; η^2^ = 0.027), the BDI-II total score (p = 0.044; η^2^ = 0.016), and the anhedonia factor score (p = 0.002; η^2^ = 0.034), but not the BSI-D score, the negative affect factor score, and the inhibition factor score. Regarding BDI-II subscales, there was a significant group difference for the somatic/affective symptom score (p = 0.045; η^2^ = 0.016), but not for the cognitive/affective symptom score (p>0.09).

**Table 3:** Vitamin D status and unadjusted and adjusted levels of depressive symptoms.

Depressive symptomatology	Deficiency	Insufficiency	Sufficiency	P-value
	25-OH D <50 nmol/l	25-OH D = 50–75 nmol/l	25-OH D > 75 nmol/l	
	n = 211, 55.5%	n = 121, 31.8%	n = 48, 12.6%	
**HADS-D scores**				
Unadjusted mean ± SD	13.13 ± 4.23 ^1^ ^,^ ^2^	11.98 ± 4.33	11.27 ± 4.47	0.006
Fully adjusted mean ± SEM	13.12 ± 0.29 ^1^ ^,^ ^2^	12.00± 0.38	11.28 ± 0.61	0.010
**BDI-II scores**				
*Total scores*				
Unadjusted mean ± SD	31.51 ±11.86 ^2^	30.10 ±11.75	26.92 ±10.04	0.044
Fully adjusted mean ± SEM	31.67 ± 0.77 ^2^	29.90 ±1.01	26.70 ± 1.62	0.023
*Cognitive/affective scores*				
Unadjusted mean ± SD	11.86 ± 0.42	11.51 ± 0.56	9.73 ± 0.89	0.095
Fully adjusted mean ± SEM	12.04 ± 0.39 ^2^	11.33 ± 0.51 ^3^	9.39 ± 0.82	0.018
*Somatic affective scores*				
Unadjusted mean ± SD	19.65 ± 0.45 ^2^	18.59 ±0.59	17.19 ± 0.94	0.045
Fully adjusted mean ± SEM	19.63 ± 0.45	18.57 ± 0.59	17.32 ± 0.95	0.078
**BSI-D scores**				
Unadjusted mean ± SD	11.84 ± 5.92	11.43 ±5.89	10.69 ± 5.22	0.442
Fully adjusted mean ± SEM	11.88 ± 0.39	11.34 ± 0.50	10.74 ± 0.81	0.422
**Negative affect factor scores**				
Unadjusted mean ± SD	0.034 ± 1.032	0.022 ± 0.956	-0.203 ± 0.957	0.321
Fully adjusted mean ± SEM	0.058 ± 0.064	-0.005 ± 0.083	-0.243 ± 0.13	0.140
**Anhedonia factor scores**				
Unadjusted mean ± SD	0.160 ± 0.978 ^1,2^	-0.160 ±0.997	-0.300 ± 0.992	0.002
Fully adjusted mean ± SEM	0.155 ± 0.066 ^1,2^	-0.157 ± 0.086	-0.288 ± 0.137	0.003
**Inhibition factor scores**				
Unadjusted mean ± SD	0.024 ± 1.004	0.010 ± 1.000	-0.128 ± 0.993	0.633
Fully adjusted mean ± SEM	0.002 ± 0.071	0.027 ± 0.093	-0.076 ± 0.149	0.837

Group comparisons were calculated with multivariate analysis of variance (unadjusted) or covariance with full adjustment for age, sex, season, skin pigmentation, obesity, elevated liver enzyme levels, elevated C-reactive protein levels, psychiatric comorbidity (anxiety disorder, posttraumatic stress disorder, somatoform disorder, eating disorder), recurrent depressive episode, medication (vitamin D supplements, number of antidepressants, use of neuroleptics, use of anticonvulsants, number of pain medications), and vitamin D assay.

^1^ significantly higher with vitamin D deficiency than vitamin D insufficiency.

^2^ significantly higher with vitamin D deficiency than vitamin D sufficiency.

^3^ significantly higher with vitamin D insufficiency than with vitamin D sufficiency.

BDI-II, Beck Depression Inventory-II; BSI-D, depression subscale of the Brief Symptom Inventory; HADS-D, depression subscale of the Hospital Anxiety and Depression Scale.

Post hoc analyses revealed that patients with vitamin D deficiency had greater levels of HADS-D symptoms than those with vitamin D deficiency (p = 0.019, Cohen's *d* = 0.27) or sufficiency (p = 0.007, *d* = 0.43). Likewise, BDI-II symptoms were higher in patients with vitamin D deficiency compared to those with sufficient vitamin D (p = 0.014, *d* = 0.42) and the same was observed for somatic/affective symptoms (p = 0.019; *d* = 0.40). Patients with vitamin D deficiency also scored higher on the anhedonia factor than those with insufficient (p = 0.005, *d* = 0.32) or sufficient (p = 0.004, *d* = 0.47) vitamin D.

#### Fully adjusted models

The significance of the results from the MANOVA analyses was maintained in the fully adjusted MANCOVA analyses. After controlling for all covariates, there were significant group effects for vitamin D status for depressive symptom scales (F_3,358_ = 4.41, p = 0.005; η^2^ = 0.036) and factor scores (F_3,358_ = 4.47, p = 0.004; η^2^ = 0.036).

As shown in Table 3, the three groups significantly differed in the HADS-D score (p = 0.010; η^2^ = 0.025), the BDI-II total score (p = 0.023; η^2^ = 0.021), and the anhedonia factor score (p = 0.003; η^2^ = 0.032), but not the BSI-D score, the negative affect factor score, and the inhibition factor score. Regarding BDI-II subscales, there was a significant group difference for the cognitive/affective symptom score (p = 0.018; η^2^ = 0.022), but not for the somatic/affective symptom score (p>0.08).

Again, post hoc analyses revealed that patients with vitamin D deficiency had greater levels of HADS-D symptoms than those with vitamin D insufficiency (p = 0.023) or sufficiency (p = 0.008); BDI-II total symptoms were also higher in patients with vitamin D deficiency compared to those with sufficient vitamin D (p = 0.007). Compared to patients with sufficient vitamin D, those with vitamin D deficiency (p = 0.005) and also those with vitamin D insufficiency (p = 0.041) had higher levels of cognitive/affective symptoms. Moreover, patients with vitamin D deficiency scored higher on the anhedonia factor than those with insufficient (p = 0.005) or sufficient (p = 0.005) vitamin D.

Several covariates showed a significant and independent association with depressive symptom scales and factor scores in the fully adjusted models (p-values <0.05; S1 Table).

#### Supplementary multivariate analyses

In an attempt to corroborate the above multivariate results, we performed three complementary analyses. Firstly, numerous physical medical conditions have been associated with both depression and low vitamin D levels and thus might confound the observed relationships. However, the significance of the relationship between vitamin D status and depressive symptomatology did not change with additional adjustment for the 22 patients with a comorbid cardiovascular, renal, or kidney disease. Secondly, overweight (not only obesity) has also been associated with low vitamin D levels. The significance of the relation between vitamin D status and depressive symptomatology was maintained, when controlling for overweight (BMI ≥25 kg/m^2^ vs. BMI < 25 kg/m^2^) instead of obesity (except for somatic/affective symptoms which turned out to be similar in patients with insufficient and sufficient vitamin D). Thirdly, the fact that two assay methods were used to assess 25-OH D could be a confounding factor even when controlling for a dummy coded variable. An interaction term “vitamin D status-by-assay method” was not significant, supporting the notion that results were not evidently affected by the use of two different 25-OH D assays.

## Discussion

We found that vitamin D deficiency was significantly associated with increased levels of depressive symptoms in patients hospitalized with a depressive episode. To our knowledge, this is the first study investigating a relation between vitamin D deficiency and a psychiatric diagnosis of depression in hospitalized patients. The findings may be important because the measurement of vitamin D levels has increasingly become routine in clinical psychiatry with the hope to offer a new therapeutic option for depression [34]. Significant functional impairment having compromised outpatient therapy was a premise for our patients to be hospitalized. Therefore, hospitalized patients are in particular need of multicomponent antidepressant therapy, making vitamin D a potentially attractive adjunct to established therapies such as antidepressant medication [7].

In terms of good clinical practice for vitamin D substitution in depression, the current literature suggests that vitamin D substitution will only benefit individuals who a) have clinically relevant depressive symptoms [16], b) are vitamin D deficient based on measured 25-OH D levels and c) will also increase their 25-OH D levels [6]. In other words, too low amounts of vitamin D (<800 I.U. daily) in those with vitamin D deficiency, as well as vitamin D substitution in those with sufficient vitamin D, will both be ineffective in depressed individuals [6]. Although we did not study an effect of vitamin D on depressive mood, our findings support the notion that, even in hospitalized clinically depressed patients, vitamin D deficiency is associated with significantly more depressive symptoms than vitamin D sufficiency after controlling for important covariates of 25-OH D levels and depression. This difference seemed considerably robust, as it was observed for both the HADS-D and BDI-II total scores. Importantly, the HADS-D excludes somatic/affective symptoms of depression, and our analysis on BDI-II subscales revealed vitamin D deficiency to be significantly related to cognitive/affective symptoms but not somatic/affective ones. Therefore, vitamin D deficiency could particularly be associated with cognitive/affective symptoms of depression, but less so with somatic/affective ones. In terms of HADS-D scores, patients with deficient vitamin D also had more depressive symptoms than those with insufficient vitamin D with, although the latter group did not differ from those with sufficient vitamin D. Somewhat different from these post hoc findings, BDI-II cognitive/affective symptom scores were higher in patients with insufficient versus sufficient vitamin D but similar in those with deficient and insufficient vitamin D. The effects sizes of the above observations were small-to-moderate, and, thus, of clinical relevance. We thus interpret that vitamin deficiency (25-OH D levels <50 nmol/l) may warrant particular clinical attention in patients hospitalized with psychiatric depression. Although this can be assumed, randomized controlled trials are needed to show that depressive symptoms can indeed be lowered in these patients through vitamin D supplementation above and beyond standard antidepressant treatment.

As a novel aspect of our study, we performed a principal component analysis on depressive symptom items from three validated and widely used depression symptom scales. We found that symptoms related to a factor reflecting “Anhedonia” were significantly increased in vitamin deficient patients compared with those having insufficient or sufficient vitamin D. In contrast, two other depressive symptom factors, we denoted with “Negative Affect” and “Inhibition”, were unrelated to vitamin D deficiency. Anhedonia is a core symptom of a depressive episode which refers to diminished pleasure from, or interest in, previously rewarding activities. A single infusion of the noncompetitive N-Methyl-D-aspartate receptor antagonist ketamine has recently been shown to rapidly improve symptoms of anhedonia in patients with major depression and treatment-resistant bipolar disorder, with an effect lasting up to three and 14 days, respectively [35,36]. Anti-hedonic effects of ketamine were mediated through changes in metabolic activity in the dorsal anterior cingulate cortex, orbitofrontal cortex, putamen, and the hippocampus [35,36]. In agreement with a potential role of vitamin D in the neurobiology of anhedonia, vitamin D receptors have also been found in the hippocampus both in the rodent and human brain [37,38]. Depressive/anhedonia-like behavior in rodents exposed to unpredictable chronic mild stress was associated with downregulated expression of the serotonin transporter and upregulated expression of vitamin D receptors both in the hippocampus [39,40]; this may suggest a compensatory mechanism through vitamin D signaling that protects from stress-induced effects on the brain resulting in anhedonia [39]. There is no approved medication to improve anhedonia and standard treatments of depression do little to alleviate symptoms of anhedonia [35,36]. Therefore, future randomized controlled trials may want to particularly test for an effect of vitamin D supplementation on anhedonia symptoms.

We expectedly found that patients who were hospitalized during the winter season, and those with a greater number of antidepressant medications, showed an increased frequency of vitamin D deficiency, which, in turn was less frequent in those taking vitamin D supplements. Controlling for these variables and for other potential confounders did not change the significant relationship between vitamin D deficiency and depressive symptoms (except for the somatic/affective symptom subscale of the BDI-II). Also, the relationship was not attenuated with adjustment for covariates that were associated with increased levels of depressive symptoms, including demographic variables, psychiatric comorbidity, medication use, and recurrent depression. This may suggest our findings on a role of vitamin D deficiency for depressive symptoms can be generalized to hospitalized patients with clinical depression who otherwise are not uniform in population characteristics.

Strengths of the current study were the sample size, allowing us to compute factor analysis with an ample number of items from three validated depression questionnaires, and the assessment of psychiatric diagnoses with standardized criteria in hospitalized patients. Limitations included the observational design with retrospectively collected data. Specifically, because the selection of study participants after measuring vitamin D may not be random, we cannot rule out a selection bias. During the study period, the knowledge of a potentially important role of vitamin D for mental health has been accumulating, so all medical staff was increasingly advised to routinely order 25-OH D levels at hospital entry. However, before this was fully implemented, 25-OH D may have been measured based on risk factors, making those included in the study different from those excluded. We were unable to obtain information on several variables that may affect depression and vitamin D levels, so residual confounding cannot be excluded. Specifically, we were unable to adjust our analyses for physical activity (both indoors and outdoors), sun exposure and the use of sunscreen, as well as for dietary sources of vitamin D. This lack of information is important in interpreting our data because patients with clinical depression generally exhibit unhealthy behaviors, psychomotor retardation, and social withdrawal, all of which might directly or indirectly influence 25-OH D levels. Additional controlling for important physical medical conditions did not alter our results in a supplementary analysis. However, in a clinical sample as ours, it is impracticable to control for all the mental and physical comorbidities which all can be associated with depression and low vitamin D levels in their own right. We did not assess depressive symptoms by a clinical interview, although the association of 25-OH D levels with depressive symptoms has been shown to be stronger when depression was assessed by diagnostic interviews compared with self-reported symptom scales [1]. Nevertheless, we applied validated psychometric instruments, such as the HADS-D, with an ability to identify depression “caseness” with a sensitivity of 82% and a specificity of 74% when applying a cut-off score ≥8 [41]. The method of vitamin D measurement changed halfway through the study. This is unfortunate as commercially available 25-OH D assays often show not the same accuracy to detect low vitamin D levels [42]. However, controlling for the two assay methods and results from a supplementary moderator analysis suggested that our findings were not evidently affected by using two assay methods, although we acknowledge that some uncertainty remains regarding this methodological issue.

## Conclusions

This study showed that vitamin D deficiency at 25-OH D levels <50 nmol/l is significantly associated with increased levels of depressive symptoms in patients hospitalized with psychiatric depression. This finding was suggested to be robust and of clinical relevance with cognitive/affective symptoms and particularly anhedonia symptoms being affected by vitamin D deficiency. Based on previous knowledge, it might be prudent to supplement this patient population with at least 800 I.U. daily of vitamin D. Whether those high in anhedonia will particularly profit from such practice, is an important question to be examined in future intervention studies.

## Supporting Information

S1 Table Significant covariates of depressive symptoms from the multivariate fully adjusted prediction models. (PDF)
