# Supplementary material for: Vitamin D Deficiency and Depressive Symptomatology in Psychiatric Patients Hospitalized with a Current Depressive Episode: A Factor Analytic Study
Source: PLoS One. 2015 Sep 23;10(9):e0138550. doi: 10.1371/journal.pone.0138550 (PMC4580407; doi:10.1371/journal.pone.0138550)
Supplement: S1 Table — (PDF) [file pone.0138550.s001.pdf]

Table S1. Significant covariates of depressive symptoms from the multivariate fully adjusted prediction models

**Variables**	**HADS-D**		**BDI-II**		**BSI-D**		**Negative affect factor**		**Anhedonia factor**	
	B±s.e.	P	B±s.e.	P	B±s.e.	P	B±s.e.	P	B±s.e.	P
Age (years)			-0.182±0.044	<0.001	-0.082±0.022	<0.001	-0.024±0.004	<0.001		
Female sex	-1.113±0.475	0.020							-0.245±0.107	0.023
Dark skin pigmentation	2.321±0.574	<0.001	3.505±1.523	0.022	2.210±0.764	0.004			0.616±0.130	<0.001
Anxiety disorder									0.227±0.111	0.042
PTSD			4.939±2.188	0.025			0.479±0.181	0.008		
Eating disorder	1.881±0.849	0.027	5.884±2.251	0.009	2.290±1.128	0.043	0.546±0.186	0.004	0.482±0.192	0.012
Antidepressants	0.930±0.328	0.005	3.033±0.870	0.001	1.131±0.436	0.010	0.220±0.072	0.002	0.249±0.074	0.001
Neuroleptic use	1.280±0.522	0.015			1.694±0.694	0.015	0.240±0.114	0.037	0.370±0.118	0.002
Recurrent depression					1.564±0.609	0.011	0.264±0.100	0.009		

Value are unstandardized coefficients B with standard error (s.e.). Prescribed drugs were entered with 3 categories for antidepressants (0, 1, 2) and two categories for neuroleptics (0, ≥1). Read table as follows (example): Patients with PTSD had a 4.939±2.188 higher BDI score than those without PTSD after all other covariates (including vitamin D status) had been taken into account.

BDI-II, Beck Depression Inventory-II; BSI-D, depression subscale of the Brief Symptom Inventory; HADS-D, depression subscale of the Hospital Anxiety and Depression Scale; PTSD, posttraumatic stress disorder
